# Correlation of Clinically-Suspected Spontaneous Bacterial Peritonitis (SBP) With Laboratory-Confirmed SBP in Portosystemic Encephalopathy Patients

**DOI:** 10.7759/cureus.31120

**Published:** 2022-11-05

**Authors:** Himayat Ullah

**Affiliations:** 1 College of Medicine, Shaqra University, Shaqra, SAU

**Keywords:** spontaneous bacterial peritonitis (sbp), clinical features, ascitic fluid neutrophil count, portosystemic (hepatic) encephalopathy, ascites

## Abstract

Background: Spontaneous bacterial peritonitis (SBP) is among the most common complications of liver cirrhosis with ascites. In the past, it was considered a potentially incurable disease, but its prognosis, though still quite poor, has much improved in the past few years. This has become possible due to early diagnosis and prompt treatment of this once-incurable complication of ascites. The main aim of this study was to know the relation between clinically suspected SBP and laboratory-confirmed SBP so that in the absence or delay in the more accurate diagnostic facilities, clinicians can start the treatment promptly based on diagnostically significant clinical findings while awaiting the most accurate diagnostic tests.

Material and methods: This study was done at the Department of Gastroenterology, Hayatabad Medical Complex, Peshawar. After ethical approval, 186 patients with classical features of SBP i.e., fever and abdominal pain and/or tenderness (clinically SBP patients), and 104 patients without these features (clinically non-SBP patients) were studied for ascitic fluid neutrophils count, as a diagnostic test for SBP.

Results: Out of 186 patients with clinically suspected SBP, 171 (91.9%) patients had laboratory-confirmed SBP and 15 (8.1%) had no SBP. Among 104 clinically non-SBP patients, 90 (86.5%) had laboratory-confirmed non-SBP, while 14 (13.5%) had SBP in laboratory studies. The sensitivity, specificity, positive predictive value, and negative predictive value of the clinical features in diagnosing SBP were 92%, 86%, 92%, and 87% respectively.

Conclusion: Clinical features diagnostic for SBP can play a vital role in early diagnosis and hence requires prompt treatment in circumstances where diagnostic laboratory tests are not available and/or are delayed.

## Introduction

Cirrhosis liver and its related complications are among the leading health issues worldwide with related mortality of approximately one million deaths per year [1]. It affects both the rich and the poor equally with the rich being more prone to obesity and alcohol-related chronic liver disease and poor to the hepatotropic viruses. Among patients with liver cirrhosis, ascites is one of the most prevalent complications. Though there are many causes of ascites, more than 75% of cases with ascites are due to portal hypertension in patients with cirrhosis liver [2]. Other causes may be hypoalbuminemia due to renal disease, primary or secondary neoplasm, infections like tuberculosis, and inflammatory diseases like systemic lupus erythematosus (SLE), etc. A vast majority of the patients with cirrhosis liver develop ascites as the disease progresses. The pathophysiology of ascites is not clearly understood but there are theories about the accumulation of ascites like the under-filling theory, over-flow theory, and the most recent one, the peripheral vasodilation theory which is the combination of both [3]. Early in the disease, there is splanchnic and peripheral vasodilation leading to underfilling. This leads to activation of the renin-angiotensin and aldosterone (RAAS) system and other neurohormones causing excessive salt water retention and hence overflowing. Moreover, decreased oncotic pressure due to hypoalbuminemia caused by cirrhosis liver is an additive effect to the whole scenario. 

Spontaneous bacterial peritonitis (SBP) is one of the most common and fatal complications of ascites in patients with cirrhosis liver. It is a bacterial infection of the ascitic fluid without any identifiable source of infection [4]. It develops in 10% to 30% of the patients with ascites undergoing routine ascitic fluid analysis [5]. The main complications of SBP are septicemia, renal failure, worsening hepatic encephalopathy, coma, and death. SBP is usually mono-bacterial with Escherichia coli being the commonest organism followed by other gram-negative rods like Klebsiella and gram-positive organisms like Streptococci and Staphylococci [6]. The pathogenesis of SBP is thought to be due to the translocation of bacteria through the gut due to bacterial overgrowth inside the small intestines, which is a common finding in patients with liver cirrhosis [7]. The common clinical features of SBP are fever with chills and abdominal pain and tenderness in patients with ascites. Other features are diarrhea, development or worsening of encephalopathy, increasing volume of ascites, and development or worsening of renal impairment [4]. Diagnosis of SBP is confirmed by the ascitic fluid neutrophil count of 250/µL (250 x 106/L) or more in the absence of any other cause like secondary peritonitis [4,8]. Ascitic fluid culture is not a sensitive test for the diagnosis of SBP as it has a 40% to 60% positivity rate.

Though the majority of the patients with SBP have classical symptoms like fever, abdominal pain, and tenderness, there are some patients with SBP who reported having no such symptoms [9]. There is not much data available on the topic that how often patients with SBP have classical clinical features (fever and abdominal pain/tenderness), and how often patients with no classical features have SBP. So, a study was designed to correlate the clinically suspected SBP with laboratory-confirmed SBP.

The objective of this study was to see how many patients with classical clinical features (fever, abdominal pain, or tenderness) of SBP have laboratory-confirmed SBP, and also to see how many patients with ascites but no classical features of SBP have laboratory-confirmed SBP.

## Materials and methods

This study was done at the Department of Gastroenterology, Hayatabad Medical Complex, Peshawar after approval from the Hospital Research and Ethical Committee, Medical Teaching Institute (MTI), Hayatabad Medical Complex, Peshawar (Ref No. 597/HEC/B&PSC/2021). A total of 186 hepatic encephalopathy patients with classical clinical features of SBP (fever and abdominal pain/tenderness) and 104 patients without these features were recruited in this study. The sample size was calculated by a standardized sample size calculator based on the global prevalence of SBP in patients with ascites [10]. The sampling technique used was non-probability convenience sampling. Inclusion criteria were all patients above 12 years of age with ascites and hepatic encephalopathy. The exclusion criteria were any patient with fever, abdominal pain, or tenderness attributable to any condition other than SBP, like secondary peritonitis, any other infective process, etc. Informed consent was obtained from all the patients and then were subjected to detailed history followed by clinical examination. The clinical criteria that were considered for the diagnosis of SBP were fever with or without chills (oral temperature > 99oF) and abdominal pain or tenderness. The laboratory diagnosis of SBP was made by a neutrophil count of ≥ 250/µL of ascitic fluid.

The data analysis was done by MS Excel (Microsoft Corp., Washington, USA) and Statistical Package for Social Sciences (SPSS), version 22 (IBM Corp., Armonk, NY, USA). Means and standard deviations were calculated for quantitative variables like age, and frequencies and percentages for categorical variables like gender, clinically diagnosed SBP, and laboratory-confirmed SBP. Sensitivity, specificity, positive predictive value, and negative predictive value of the clinical features of SBP (fever, abdominal pain/tenderness) for diagnosing SBP were also calculated. Tables and graphs were used for presenting the data.

## Results

The odds ratio calculated the significance of the relation between classical clinical features of SBP with confirmed SBP; it was found to be 73.3 with a 95% confidence interval which indicates the strength of the relationship between the two. In this study, a total of 290 patients with ascites and portosystemic encephalopathy were assessed for clinical features of SBP. There were 175 (60.3%) males and 115 (39.7%) females with a male-to-female ratio of 1.52 in the study. Gender distribution is shown in Table 1.

**Table 1:** Gender Distribution of Patients with Ascites

gender statistics	male	female	total
frequency	175	115	290
percentages	60.3%	39.7%	100%

Among these, 37 (12.8%) patients were between 13 to 40 years, 153 (52.8%) patients were between 41 to 60 years, and 100 (34.4%) patients were above 60 years of age. Most of the patients were above 40 years of age i.e., 153 (87.3%) patients and the mean (SD) age of the patients was 54.71 (17.11) (Table 2).

**Table 2:** Age Distribution in Patients with Ascites

Age (Years)	Frequency	Percentage	Mean Age	SD
13 – 40	37	12.8%	26.70	10.749
41 – 60	153	52.8%	49.50	6.696
> 60	100	34.5%	73.04	8.327
TOTAL	290	100.0%	54.71	17.110

Among these patients, 186 had the classical clinical features of SBP, and 104 patients did not have the clinical features. Table 3 shows the segregation of these patients based on clinically suspected SBP and non-SBP patients, and lab-confirmed SBP and non-SBP patients. Out of 186 patients with classical features of SBP, 171 (91.9%) patients were confirmed to have SBP, and 15 (8.1%) patients were confirmed to be non-SBP on the basis of ascitic fluid neutrophil count ≥ 250/ μL (the laboratory diagnostic criteria). Similarly, out of 104 patients with ascites but without the classical clinical features of SBP, 90 (86.5%) patients were confirmed to be non-SBP whereas 14 (13.5%) patients were found to be fulfilling the laboratory criteria for the diagnosis of SBP, as shown in Table 3.

**Table 3:** Correlation of Clinically-suspected SBP and Laboratory-confirmed SBP SBP: spontaneous bacterial peritonitis.

clinical features	frequency	percentage
clinically SBP	186	100%
lab confirmed SBP	171	91.9%
lab confirmed non-SBP	15	8.1%
clinically non-SBP	104	100%
lab confirmed non-SBP	90	86.5%
lab confirmed SBP	14	13.5%

**Figure 1:**
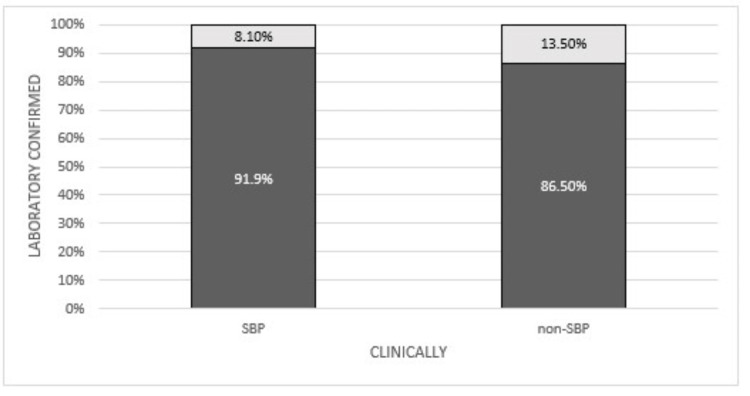
Correlation of Clinically SBP and Clinically Non-SBP Patients with Laboratory-confirmed SBP and Laboratory-confirmed Non-SBP Patients SBP: spontaneous bacterial peritonitis.

In Figure 1, patients with clinical features of SBP and those without were plotted against the patients with laboratory-confirmed SBP and non-SBP patients.

Based on the presence of clinical features, true positives and false positives were identified, whereas, on the basis of the absence of clinical features of SBP, true negatives and false negatives were identified through cross-tabulation. Sensitivity, specificity, positive predictive value, and negative predictive value were calculated by using their formulas, which came out to be 92%, 86%, 92%, and 87% respectively, as shown in Table 4. 

**Table 4:** Clinical SBP Cross-tabulation with Laboratory Confirmed SBP Showing Sensitivity, Specificity, Positive and Negative Predictive Values of the Classical Features in Diagnosis of SBP SBP: spontaneous bacterial peritonitis.

				Lab-confirmed SBP			Total		
				No		yes			
SBP Clinically		No	Count	90		14	104		
			% Within Lab-confirmed SBP	85.7%		7.6%	35.9%		
		yes	Count	15		171	186		
			% Within Lab-confirmed SBP	14.3%		92.4%	64.1%		
Total			Count	105		185	290		
			% Within Lab-confirmed SBP	100.0%		100.0%	100.0%		
Sensitivity	True Positive/ (True positive + False Negative)				171/ (171 + 14)			92%	
Specificity	True Negative/ (True Negative + False Positive)				90/ (90+ 15)			86%	
Positive Predictive value	True Positive/ (True Positive + False Positive)				171/ (171 + 15)			92%	
Negative Predictive value	True Negative/ (True Negative + False Negative)				90/ (90 + 14)			87%	

The odds ratio calculated for the significance of the relation of classical clinical features of SBP with the confirmed SBP and found to be 73.3 with 95% confidence interval which indicates the strength of the relationship between the two.

## Discussion

In this study, we have established the diagnostic accuracy of the classical features i.e., fever, and abdominal pain and/or tenderness, of SBP in patients with ascites and hepatic encephalopathy. There are several nonspecific clinical features associated with SBP like fever with chills, abdominal pain, and tenderness, development of icterus, increasing ascitic fluid volume, development and/or worsening of hepatic encephalopathy, worsening of renal functions, etc [4]. Among these fever with chills and abdominal pain and/or tenderness are the most common ones [11,12]. Fever with/without chills and abdominal pain and/or tenderness are the two classical features of SBP and have been proven to be the most important and productive for the diagnosis of SBP. In one of his studies, Chinnock et al. found that fever in a patient with suspicion of SBP had 81% specificity and the absence of abdominal pain in these patients was 94% sensitive in ruling out SBP [13,14]. In their recent article on SBP, Ameer et al. mentioned different clinical features of SBP including fever, abdominal pain, worsening of hepatic encephalopathy, diarrhea, etc. but fever and abdominal pain are the most important ones [15]. They added that cirrhotic patients are usually hypothermic so a rise in temperature in these patients is a very sensitive finding if SBP is suspected.

Although different biochemical and hematological tests can be of significance in the diagnosis of SBP, like raised bilirubin, raised PT, raised CRP levels, leukocytosis, etc., for laboratory diagnosis of SBP, the ascitic fluid neutrophil count was used in this study, which is most accurate in the diagnosis of SBP [16,17]. Shi et al. in their study enumerated that serum creatinine more than 79.5 μmol/L, prothrombin time above 22.9, serum bilirubin above 63.5 μmol/L, and WBCs count above 6 x 106/L have predictive value in diagnosing SBP in suspected patients [17]. Similarly, ascitic fluid appearance can also be of diagnostic importance in SBP [18]. Ascitic fluid culture is specific for diagnosing SBP but less sensitive with 40% to 60% of false negative cases in some studies [19,20]. Although ascitic fluid leukocyte count more than or equal to 500/μL can be used for the diagnosis of SBP, ascitic fluid neutrophil count above or equal to 250/μL is a more sensitive predictor. Ameer et al., in their article, stated, that ascitic fluid leukocyte count ≥ 500/μL is 86% sensitive and 98% specific while ascitic fluid neutrophil count ≥ 250/ μL is 93% sensitive but 94% specific [15].

Although several clinical features have been enumerated in different studies mentioning fever and abdominal pain/tenderness as the most sensitive and specific symptoms in suspected SBP patients, their accuracy in diagnosing SBP was questionable and a point of debate. Few of these studies have mentioned the sensitivity and specificity of each of these symptoms separately but the combined sensitivity, specificity, and predictive values of the most prevalent clinical features (fever with/without chills and abdominal pain and/or tenderness) when taken collectively in diagnosing SBP in suspected patients, was not studied much. SBP is a very serious complication of liver cirrhosis and ascites. It is the most frequent infection in patients with liver cirrhosis and has a very high mortality, up to 40% in some studies [21,22]. This can reach 80% if the treatment is delayed and the patient develops septic shock [23]. The mortality rate is even higher in advanced disease as in one of their articles Cohen et al. found that SBP in advanced liver cirrhosis can go unnoticed and can have a very high mortality of up to 93% [24]. Early diagnosis of SBP followed by prompt treatment can be vital in such patients as supported by different studies. Orman et al. concluded from their study that early diagnostic paracentesis and treatment can decrease in-hospital as well as three months mortality and also shorten hospital stay [25]. Similar findings were reported by Kim et al. in their study [26]. Findings from these studies clearly indicate how vital the early diagnosis and prompt treatment of SBP is, in order to decrease the mortality and morbidity of this lethal complication of ascites. Currently, ascitic fluid neutrophil count is the single best test for the diagnosis of SBP, but is invasive and needs expertise and laboratory resources. The idea behind this study was, to identify the group of clinical features that has the highest diagnostic value and accuracy so that in the circumstances where ascitic fluid analysis is not available or delayed, the treatment of SBP can be started on the basis of these features while awaiting the more accurate diagnostic tests.

One of the main limitations of this study was that other less frequent clinical features of SBP like icterus, worsening encephalopathy, rise in the ascitic fluid volume, etc. were not studied, and hence the additive effect of these features is still food for future research.

## Conclusions

SBP is one of the most frequent and feared complications of ascites and cirrhosis liver. Once considered an incurable disease, the mortality, as well as the morbidity of SBP, have been much improved in recent times due to early diagnosis and proper management. Though diagnostic paracentesis is the gold standard for the diagnosis of SBP, non-invasive methods like the presence of classical clinical features (fever, abdominal pain, and/or tenderness) of SBP can play a diagnostic role whenever there is difficulty or delay in diagnostic paracentesis. 
